# 辅助化疗在早期可手术非小细胞肺癌治疗中的地位

**DOI:** 10.3779/j.issn.1009-3419.2011.03.15

**Published:** 2011-03-20

**Authors:** 相涛 闫, 辉 朱, 慧娟 王, 启鸣 王, 鹏 李, 智勇 马

**Affiliations:** 450000 郑州，河南省肿瘤医院肿瘤内科 Depatment of Medical Oncology, Henan Tumor Hosipital, Zhengzhou 450000, China

**Keywords:** 肺肿瘤, 辅助化疗, 靶向治疗, Lung neoplasms, Adjuvant chemotherapy, Targeted therapy

## Abstract

肺癌患者术后复发转移是临床治疗失败的主要原因，因此亟待一种更系统的治疗手段来完善患者的治疗计划，以降低复发率、延长生存期。辅助化疗（包括新辅助化疗、术后辅助化疗、靶向药物辅助化疗）应运而生。本文就辅助治疗领域的研究进展进行综述。

## Abstract (en)

Postoperative recurrence is the main cause of primary treatment failure in the resectable non-small cell lung cancer (NSCLC). Systematic treatments should be taken to decrease the recurrence. Adjuvant chemotherapy, including neoadjuvant chemotherapy, postoperative adjuvant chemotherapy and targeted therapy, is a widely used in this area. In this review, we summarize the clinical trials and meta-analysis related to the adjuvant chemotherapy and targeted therapy in patients with early stage NSCLC.

在过去的十几年内，由于放疗和手术技术的进步、新化疗方案和靶向药物的不断出现以及二线化疗的进步和维持治疗的开展，非小细胞肺癌（non-small cell lung cancer, NSCLC）的治疗有了很大的进展，但同时肺癌相关的癌性死亡率仍排在前列^[1]^。2009年美国的肺癌相关死亡人数为159, 390例，其中NSCLC占87%^[2]^。因此还需要一种更系统、有效的治疗策略来降低肺癌的死亡率和复发率。医学工作者很早就对辅助化疗（包括新辅助化疗、术后辅助化疗及靶向药物辅助化疗）寄予了很深的期望。2000年国际肺癌辅助治疗试验（International Adjuvant Lung Cancer Trial, IALT）的研究结果证实术后辅助化疗能改善早期可手术NSCLC的预后。目前手术联合术后含铂方案化疗已经成为早期可手术NSCLC患者的标准治疗策略。多年来术前新辅助化疗也相继有研究和报道，但是它的应用能否给肺癌患者的生存带来更多的益处还无明确定论。靶向治疗在近些年有迅速的发展，最有代表性的药物为表皮生长因子受体酪氨酸激酶抑制剂（epidermal growth factor receptor tyrosine kinase inhibitor, EGFR-TKI）类药物，不管是临床疗效还是不良反应方面都不逊色于传统的化疗，为肺癌的治疗带来了新的方法和希望。本文对近年来有关肺癌辅助化疗领域的重要临床研究进展进行了回顾综述，以期为早期可手术NSCLC的辅助化疗提供更多的依据，同时也为进一步的临床研究提供参考。

## 1 术前新辅助化疗能否获益

到目前为止，尚没有确切的数据证实术前新辅助化疗能给早期肺癌患者的生存带来获益。但是随着进一步的深入研究，术前诱导化疗已经开始在生存方面崭露头角。NATCH Ⅲ期临床试验^[3]^正是基于此种想法而设计，截至2007年共入组624例早期（肿瘤直径 > 2 cm的Ⅰ期、Ⅱ期、T3N1）NSCLC患者，有效统计病例数为582例。病例随机分组进行单纯的手术切除，或3周期术前PC（紫杉醇+卡铂）方案化疗，或手术+术后3周期PC化疗。三组的5年无进展生存率（progression free survival, PFS）分别为：单独手术组39%，术前化疗组40.5%，术后化疗组39.3%。三组的中位无病生存时间（disease free survival, DFS）分别为28个月，32个月和24个月（*P*=0.71）。探索性分析表明术前化疗组中出现影像学反应的患者中位DFS似乎更长（62个月，*P*=0.09），研究结论是三组的DFS没有统计学差异。后来的IFCT0002研究^[4]^也同样得到了阴性的结果，这项试验的主要研究目的为比较完全术前化疗组（所有化疗周期均在术前进行）和围手术期化疗组之间的生存差异。共入组528例Ⅰa-Ⅱ期可切除的NSCLC患者，随机分为术前化疗序贯手术组和术前化疗序贯手术、再序贯化疗组，根据化疗方案（吉西他滨联合顺铂/紫杉醇联合卡铂）的不同再分为2组。完全术前化疗组和围手术期化疗组的3年总生存率分别为75.1%和79.5%（*P*=0.96）。术前完成全部化疗计划的患者依从性更高，但是两组的生存无差异。亚组分析表明围手术期行吉西他滨为基础的化疗会使鳞癌患者受益更多，而以紫杉醇为基础的术前新辅助化疗可能会使非鳞癌患者获益更多。本试验进行了β微管蛋白（bTubⅢ）的免疫组化染色情况亚组分析，结果显示β微管蛋白阳性表达的部分患者不能从术前新辅助化疗中获益^[5, 6]^。排除这部分人群后术前新辅助化疗的结果是否会有所改观，我们目前尚不能得出肯定的结论。S9900试验^[7]^也是早期进行的一项有关术前新辅助化疗的临床试验，这项研究的主要目的为确定紫杉醇联合卡铂（PC）方案诱导化疗对比单纯手术是否能提高患者的生存。因为术后辅助化疗地位的确立，该试验在2007年4月提前终止。试验结果表明随访5年内，PC方案诱导化疗组的PFS和总生存略有优势，但这种差异不明显。2010年美国临床肿瘤学（American Society of Clinical Oncology, ASCO）年会上发表了一个在法国进行的随访长达10年的新辅助化疗临床试验^[8]^。这项研究早在2002就首次在临床肿瘤学（JCO）杂志中提出。中位随访13.8年，单独手术组和术前新辅助化疗组的10年无进展生存率分别为16.4%和24.5%（HR=0.78, *P* = 0.031），术前化疗组的无病生存延长。两组的10年总生存率（overall survival, OS）分别为20.8%和29.4%（HR=0.82, *P*=0.12）。虽然差异不明显，但术前化疗的长期生存（10年）有8%的获益，另外术前化疗不增加非肿瘤死亡。探索性分析表明术前化疗增加行肺叶切除术患者的生存率，但全肺切除患者不同。这是目前为止随访时间最长的一项关于术前新辅助化疗的临床试验，同其他早期的临床试验相比，其结果有更鼓舞人的一面。以上数据可以看出，我们对于术前新辅助化疗的了解还是相对贫乏，泛人群的研究显然不足以让我们信服其临床疗效。这些临床研究的选择人群是否合适存在疑问，这个领域还需要进一步的个体化研究。众所周知对于一些分期相对较晚（比如Ⅲ期）的患者术前进行诱导治疗，与更早期别的患者相比获益情况是不一样的。从治疗的角度讲还存在另外一个问题，新辅助化疗模式应该怎样设定更合适，包括化疗的给药时间、给药量以及分期的影响等。这些悬而未决的问题仍值得广大的学者进一步去探讨。

## 2 术后辅助治疗获益的相关临床证据

在第三代化疗药物面世之前，Ⅰ-Ⅲa期NSCLC的标准治疗为单独的手术切除。人们首次尝试把术后辅助治疗应用于临床是在上个世纪60年代，应用的药物为环磷酰胺。这项举动并没有掀起多大的波澜。上个世纪80年代，顺铂的加入为临床内科化疗注入了新的活力。随着顺铂在临床的广泛应用，众多的学者开始设计新的临床试验。1995年非小细胞肺癌协作组（non-small Cell Lung Cancer Collaborative Group, NSCLCCG）进行了一个包括52项临床研究的的荟萃分析^[9]^，当然这些临床研究中既有含铂化疗方案，又包括了非含铂方案，得出的结论是术后辅助化疗长期使用烷化剂对患者生存无获益，相对死亡危险比增加15%，5年生存率降低5%；术后进行含铂方案辅助化疗较单独手术相比，死亡风险比为0.87，相对死亡风险减少13%；2年、5年内化疗的绝对获益率分别为3%和5%，但这种差异无统计学意义（*P*=0.08）。

1995年后，受到NSCLCCG荟萃分析的启发，人们进行了更多与NSCLC术后辅助治疗相关的荟萃分析和临床试验。其中发表的ALPI临床研究（Adjuvant Lung Project Italy Trial）^[10]^是一个大样本的术后辅助化疗的临床试验，入组人数达1, 209例，包括了Ⅰ-Ⅲa期的术后NSCLC患者，术后随机分组进行丝裂霉素、长春酰胺、顺铂联合化疗或进行临床观察。中位随访64.5个月，两组的OS没有差异（HR=0.96, 95%CI: 0.81-1.13, *P*=0.589），PFS亦是如此（HR=0.89, 95%CI: 0.76-1.03, *P*=0.128），多因素分析只发现疾病分期和性别与生存相关。

同时在英国进行的另外一项临床研究（Big Lung Trial, BLT）^[11]^也得到了阴性的结果。这项临床研究也是基于顺铂为基础的术后联合方案化疗，共入组了381例Ⅰ-Ⅲa期的患者，其中化疗组有3%的患者只接受了术前的辅助化疗，而另外97%的患者进行了术后的辅助化疗。中位随访34.6个月，两组的OS（HR=1.02, 95%CI: 0.77-1.35, *P*=0.90）和PFS（HR=0.97, 95%CI: 0.74-1.26, *P*=0.81）均没有统计学差异。

2003年，IALT^[12]^初步结果证实术后辅助化疗能使Ⅰ期-Ⅲ期的术后NSCLC患者生存有4.1%的改善。这个研究结果的公布有划时代的意义，为后来术后辅助化疗的发展与成熟奠定了不可或缺的基础。该实验共入组1, 867例Ⅰ期-Ⅲ期的术后NSCLC患者，术后随机分组进行顺铂为基础的联合方案（包括长春碱类和VP-16）化疗或进行临床观察，其中辅助化疗组有大约1/4的患者进行了术后的放疗，中位随访56个月。首次研究结果显示术后化疗组对比观察组5年生存率改善（44.5% *vs* 40.4%, HR=0.86, 95%CI: 0.76-0.98, *P* < 0.03），化疗组的5年DFS亦有优势（39.4% *vs* 34.3%, HR=0.83, 95%CI: 0.74-0.94, *P* < 0.003），其中有7例患者（0.8%）死于化疗相关的毒性反应。

试验的长期随访数据在2008年的ASCO年会上进行了进一步的报道，截至2005年9月1日中位随访时间为7.5年。结果显示辅助化疗组的生存优势不明显（HR=0.91, 95%CI: 0.81-1.02, *P*=0.10），而在DFS方面（HR=0.88, 95% CI: 0.78-0.98, *P*=0.02）显示出了持续的获益。试验把前5年和后5年随访数据进行分析比较，结果显示两个时间段的总生存情况有统计学差异，而本试验的最终结果仅能证实术后辅助化疗能够改善术后5年的生存情况，而5年以后术后辅助化疗对延长生存的贡献越来越小^[13]^。

IALT试验^[14]^是人们在辅助治疗领域收获的开端，随后在加拿大进行的JBR-10临床研究也得到了预料中的结果。共入组482例患者，包括了Ⅰb期（T2N0）或Ⅱ期（T1N1或T2N1）的术后NSCLC。术后随机分组进行长春瑞滨联合顺铂化疗和观察对照。中期分析显示，中位随访5年，与对照组相比术后辅助化疗组的OS明显改善（94个月*vs* 73个月，HR=0.69，95%CI：0.52-0.91，*P*=0.04），5年生存获益率有15%的改善（69% *vs* 54%, *P*=0.03），化疗组没有观察到无病生存时间，对照组为46.7个月（*P* < 0.001）。这是第一个对所有患者采用第三代化疗药物的临床试验，它也是迄今为止术后辅助化疗生存获益最高的报道。但是本研究的亚组分析表明，肿瘤直径 < 4 cm的Ⅰb期患者是不获益的。目前中位随访9.3年的试验最新数据^[15]^显示长期随访仍存在生存获益（HR=0.78, 95%CI: 0.61-0.99, *P*=0.04）。亚组分析表明这种获益局限于有淋巴结转移（N1）的患者，Ⅰb期肿瘤直径≥4 cm与获益相关（*P*=0.022），直径 < 4 cm者不获益（HR=1.73, *P*=0.06），辅助化疗明显降低肺癌死亡风险（HR=0.73, 95%CI: 0.55-0.97, *P*=0.03），观察组的肺癌相关死亡风险增高（*P*=0.02），治疗组中其它原因引起的死亡和第二肿瘤引起的死亡比率无差异。

我们似乎已经可以接受术后辅助化疗能够获益这一结论，但是争论的焦点转移到了不同分期和不同药物之间获益是否有差别。美国癌症和白血病B组（Cancer and Leukemia Group B）的CALGB9633临床试验^[16]^针对于Ⅰb期的NSCLC术后辅助化疗是否能够获益做了进一步的临床观察分析。试验共入组344例Ⅰb期NSCLC患者，随机分组接受紫杉醇联合卡铂的联合化疗或观察。中位随访34个月，化疗组的4年OS改善率达12%，较对照组明显延长（*P*=0.028）。中位随访74个月，化疗组和观察组的5年OS分别为60%和58%（HR=0.83, 90%CI: 0.64-1.08, *P*=0.125）。探索性分析表明肿瘤直径 > 4 cm和辅助化疗获益相关（HR=0.69, 90%CI: 0.48-0.99, *P*=0.043）。

ANITA（Adjuvant Navelbine International Trialist Association）临床试验^[17]^是一项与JBR-10相似的临床试验。ANITA试验共入组840例Ⅰb期-Ⅲa期的术后NSCLC患者，随机分组进行长春瑞滨联合顺铂（NP）方案辅助化疗或临床观察，术后放疗可选。中位随访76个月，两组的中位生存时间分别为65.7个月和43.7个月，化疗组的死亡风险明显下降（HR=0.80, 95%CI: 0.66-0.96, *P*=0.017）。化疗组的5年总生存率改善8.6%，而且这种获益在随访7年时持续存在，但是亚组分析表明这种生存获益仅限于Ⅱ期、Ⅲa期，Ⅰb期患者不获益。

LACE（The Lung Adjuvant Cisplatin Evaluation）荟萃分析^[18]^以所提及到的以上临床试验为基础进行了进一步的汇总分析。对不同分期和不同化疗方案之间获益是否有差异进行了解答。结果显示Ⅱ期和Ⅲ期获益明显，Ⅰb期获益不明显，而Ⅰa期不获益；不同化疗药物之间（包括长春碱类、VP-16）生存获益无统计学差异（*P*=0.26）；不同年龄层（< 69岁、65-69岁、 > 70岁）生存获益亦无统计学差异（*P*=0.29）。最近报道的一项临床试验^[19]^为术后随机分组采用长春瑞滨联合顺铂（NP）或多西他赛联合顺铂（DP）化疗，初步结果表明中位随访20个月，DP组和NP组的临床疗效和安全性相当，进一步的临床随访还在进行中。另外，一些比较新的第三代化疗药物（如培美曲塞）也被美国国家综合癌症网（National Comprehensive Cancer Network, NCCN）和ASCO推荐用于术后联合顺铂辅助化疗，但这两个指南中均没有提及到其所依据的文献数据。笔者在此认为可能是这两种药物在晚期NSCLC治疗中的成功被人们理所当然的认为术后联合铂类药物辅助化疗亦然有效。

日本早在上个世纪80年代就设计进行了一些关于肺癌术后辅助化疗的临床试验，其中最有影响力的为WJSG Ⅱ（The West Japan Study Group for Lung Cancer Surgery Ⅱ）研究。该研究共入组323例Ⅰ-Ⅲ期术后NSCLC患者，随机分组进行优福定（uracil-tegafur, UFT）单药、长春地辛联合顺铂化疗后序贯UFT或临床观察。三组的5年生存率分别为64.1%、60.6%和49%（*P*=0.044），术后UFT单药辅助化疗生存最佳。另外一项有关UFT的临床研究（The Japan Lung Cancer Reserch Group Study, JLCRG）^[20]^显示，术后Ⅰ期肺腺癌患者UFT口服2年对比临床观察，5年总生存分别为88%和85%（HR=0.71, 95%CI: 0.52-0.98, *P*=0.04），T2患者中这种优势更明显（*P*=0.005）。后来的有关UFT的荟萃分析^[21]^也进一步证实，早期可手术的NSCLC术后联合UFT辅助化疗能改善生存。这些试验的结果与欧美研究有相似之处，又有不同之处，其中的差异可能与人种、地域不同有关，确切的答案还值得我们进一步去探讨。

术后辅助化疗的临床疗效目前已经是深入人心，但依然有一些遗留的问题值得继续深思。随着时间的推移，辅助化疗的获益在逐渐降低。这种降低应归因于什么因素，是化疗剂量的不足、化疗持续时间不够长，还是患者本身就存在的先天易感性，到目前为止我们还不能回答这些问题。2009年7月国际肺癌研究学会（International Association for Study of Lung Cancer, IASLC）公布了新的肺癌TNM分期系统，这个新的分期会对辅助治疗产生一些影响，如在新的分期中T2bN0M0（肿瘤直径 > 7 cm）由Ⅰb期改为Ⅱa期，而上面的临床试验提到的肿瘤直径 > 4 cm的Ⅰb期患者在新的分期中多数就变成了Ⅱa期，Ⅱa期患者进行术后的辅助化疗是推荐的。

## 3 靶向治疗在辅助治疗中的地位

目前EGFR已经成为NSCLC靶向治疗的确定性靶点。EGFR-TKI类药物厄洛替尼和吉非替尼已经成为铂类为基础的化疗后的标准二线治疗。在一项国际多中心随机Ⅲ期临床试验中^[22]^，单药吉非替尼对比PC方案一线治疗晚期NSCLC的结果显示，吉非替尼组的PFS优于化疗组（HR=0.74, 95%CI: 0.65-0.85, *P* < 0.001）。亚组分析表明EGFR突变阳性的NSCLC患者中吉非替尼组的PFS明显优于化疗组（HR= 0.48, 95%CI: 0.36-0.64, *P* < 0.001）。所以在*EGFR*基因突变阳性的晚期NSCLC的治疗中，不管是一线还是二线应用EGFR-TKI类药物我们都有充分的依据。那么靶向治疗能否用于早期可手术NSCLC的辅助治疗中，最新公布的临床试验NCIC CTG BR 19^[23]^结果给了我们一些提示。试验计划共入组Ⅰb-Ⅲa期术后NSCLC患者1, 160例，目前实际入组503例，术后随机分组进行吉非替尼250 mg或安慰剂治疗，疗程为2年。中位随访4.7年，两组的中位OS和中位PFS均没有统计学差异（*P*=0.14, *P*=0.15），多因素亚组分析表明吉非替尼组中不抽烟和肿瘤直径 > 4 cm者生存获益明显。本实验虽然得到了阴性结果，但是多因素分析中不抽烟是生存获益相关的因素，这也提示了如果对患者进行进一步的临床选择可能结果会有所不同。另外笔者认为此项试验还是存在一些问题的，这项试验于2002年开始启动，2003年后术后辅助化疗开始被广泛采用，因此研究者陷入了两难的境地，对部分入组患者也采用了联合术后辅助化疗的策略，而直到2005年试验才不得不终止，由此推断这项试验存在一定缺陷。在后期的数据统计分析中我们也没有看到有关术后辅助化疗的数据，所以这项试验的结果并不足以让我们信服。一项在2010年ASCO年会上提出的NCT00600587临床试验^[24]^，它是一项以靶向治疗作为术前新辅助化疗的前瞻性研究，计划入组61例Ⅲa-N2期可切除的NSCLC患者，实际入组13例，随机分组进行厄洛替尼新辅助化疗（150 mg，每天一次口服，共42天）或吉西他滨联合卡铂的联合化疗，试验目前还在入组阶段，我们也期待着其初步结果的公布。

## 4 展望

辅助化疗是从一个独特的视角去分析和完善早期可手术NSCLC的治疗，它的临床疗效已经被我们所证实和接受，在个体化的治疗原则下还需要进一步努力寻找和发现更好的运用这一治疗手段的方法。目前已经有研究者尝试在原有化疗方案的基础上加用其它一些药物进行术后治疗（如重组人血管内皮抑制素^[25]^等），这项研究的中期分析显示采用NP联合重组人血管内皮抑制素治疗的患者与接受单纯NP治疗的患者相比，前者有较长的中位无病生存期（21.9个月*vs* 18个月，*P*=0.325, 7）。虽然目前尚无统计学差异，但人们对其最终结果寄予了厚望。而靶向药物作为辅助化疗的临床试验尚缺乏成熟的数据，不过我们依旧期待着其能给辅助化疗注入新的活力。
